# Heterogeneity in Kv2 Channel Expression Shapes Action Potential Characteristics and Firing Patterns in CA1 versus CA2 Hippocampal Pyramidal Neurons

**DOI:** 10.1523/ENEURO.0267-17.2017

**Published:** 2017-08-24

**Authors:** Stephanie Palacio, Vivien Chevaleyre, David H. Brann, Karl D. Murray, Rebecca A. Piskorowski, James S. Trimmer

**Affiliations:** 1 Department of Neurobiology, Physiology, and Behavior, University of California, Davis, CA 95616; 2 INSERM U894, Team Synaptic Plasticity and Neural Networks, Université Paris Descartes, Paris 75014, France; 3 Department of Neuroscience, Columbia University, New York, NY 10032; 4 Center for Neuroscience, University of California, Davis, CA 95616; 5 Department of Physiology and Membrane Biology, University of California Davis School of Medicine, Davis, CA 95616

**Keywords:** Kv2 channel, hippocampus, CA1, CA2, AMIGO-1, RGS14

## Abstract

The CA1 region of the hippocampus plays a critical role in spatial and contextual memory, and has well-established circuitry, function and plasticity. In contrast, the properties of the flanking CA2 pyramidal neurons (PNs), important for social memory, and lacking CA1-like plasticity, remain relatively understudied. In particular, little is known regarding the expression of voltage-gated K^+^ (Kv) channels and the contribution of these channels to the distinct properties of intrinsic excitability, action potential (AP) waveform, firing patterns and neurotransmission between CA1 and CA2 PNs. In the present study, we used multiplex fluorescence immunolabeling of mouse brain sections, and whole-cell recordings in acute mouse brain slices, to define the role of heterogeneous expression of Kv2 family Kv channels in CA1 versus CA2 pyramidal cell excitability. Our results show that the somatodendritic delayed rectifier Kv channel subunits Kv2.1, Kv2.2, and their auxiliary subunit AMIGO-1 have region-specific differences in expression in PNs, with the highest expression levels in CA1, a sharp decrease at the CA1-CA2 boundary, and significantly reduced levels in CA2 neurons. PNs in CA1 exhibit a robust contribution of Guangxitoxin-1E-sensitive Kv2-based delayed rectifier current to AP shape and after-hyperpolarization potential (AHP) relative to that seen in CA2 PNs. Our results indicate that robust Kv2 channel expression confers a distinct pattern of intrinsic excitability to CA1 PNs, potentially contributing to their different roles in hippocampal network function.

## Significance Statement

CA1 and CA2 pyramidal neurons (PNs) play distinct roles in hippocampal network function. Determining the molecular mechanisms that regulate excitability of CA1 versus CA2 PNs is important in understanding their distinct plasticity, and their different roles in spatial and contextual versus social memory, respectively. Here, we show that specific characteristics of action potential (AP) firing properties in CA1 versus CA2 PNs can be attributed to voltage-gated K^+^ (Kv)2 channels, with higher expression levels and functional contributions in CA1 versus CA2. Our results suggest that Kv2 channel expression is an important determinant of specific aspects of AP firing properties in CA1 versus CA2 PNs, and that regulation of membrane excitability by Kv2 channels may contribute to the robust synaptic plasticity of CA1 PNs.

## Introduction

Possibly the most studied neurons in the brain, CA1 pyramidal neurons (PNs) receive and integrate input from area CA3 along their dendritic arbor, and then communicate information to cortical and subcortical regions. The CA1 region is a major component of the hippocampal tri-synaptic circuit and allows the formation of spatial and contextual memory. In contrast, the role of region CA2 has been more difficult to decipher. *In vivo* studies have demonstrated that CA2 PNs do not encode spatial information in the same way as CA3 and CA1 (Mankin et al., 2015). CA2 PNs are necessary for social recognition memory (Hitti and Siegelbaum, 2014; Stevenson and Caldwell, 2014) and act to control hippocampal excitability on a global scale (Boehringer et al., 2017). Moreover, CA2 PNs form the crux of a hippocampal-wide network that encodes spatial information during immobility (Kay et al., 2016). Molecular profiling studies have identified distinct mRNA expression patterns across the CA regions in the hippocampus, clearly demonstrating the sharp border that exists between CA1 and CA2 that is detectible with a growing number of molecular markers (Talley et al., 2001; Lein et al., 2004; Lein et al., 2005). However, little is known of the expression levels of voltage-gated K^+^ (Kv) channels, the key determinants of intrinsic excitability, action potential (AP) wave form, firing patterns, and neurotransmission between CA1 and CA2 PNs.

The Kv2 family of Kv channels, which includes the Kv2.1 and Kv2.2 principal or α subunits, and the AMIGO-1 auxiliary subunit, are abundantly expressed in the soma, proximal dendrites and axon initial segment of many types of brain neurons (Trimmer, 1991; Maletic-Savatic et al., 1995; Kuja-Panula et al., 2003; Rhodes et al., 2004; King et al., 2014; Mandikian et al., 2014; Bishop et al., 2015). In the hippocampus, CA1 PNs express high levels of Kv2 channels (Maletic-Savatic et al., 1995; Rhodes et al., 2004; Speca et al., 2014; Bishop et al., 2015), which underlie ∼60–80% of the delayed rectifier current recorded from PN somata (Murakoshi and Trimmer, 1999; Du et al., 2000; Liu and Bean, 2014). In CA1 PNs, studies using antisense oligonucleotide knockdown approaches (Du et al., 2000) or employing the selective Kv2 blocking neurotoxin Guangxitoxin-1E or GxTX (Liu and Bean, 2014) showed that Kv2 channels contribute to controlling the excitability of CA1 PNs. These channels, with their relatively slow activation kinetics (Guan et al., 2007), regulate repetitive firing, AP width and trough voltage after a spike. The regulation of membrane excitability by these channels likely contributes to the robust synaptic plasticity of CA1 PNs.

Clearly, regions CA1 and CA2 are distinct, with contrasting molecular compositions and roles in hippocampal function. However, many questions remain concerning the significance of the distinct molecular profiles across areas CA1 and CA2. Given the dynamic regulation of Kv2 channels, and their pertinence in disease (Torkamani et al., 2014; Thiffault et al., 2015) and their influence on neuronal and behavioral excitability (Speca et al., 2014), a detailed understanding of their expression across regions of the hippocampus, and how this impacts cellular excitability, would further our understanding of hippocampal function. In the present study we used antibodies against the CA2 PN marker RGS14, an accepted molecular marker for CA2 PNs (Lee et al., 2010; Kohara et al., 2014), and genetically encoded *Amigo-2/Cre* mice expressing GFP in CA2 PNs (Hitti and Siegelbaum, 2014), with a panel of highly validated Kv2 channel subunit antibodies in multiplex fluorescence immunohistochemistry experiments on mouse brain sections to determine the cellular expression of Kv2 channel principal and auxiliary subunit polypeptides across hippocampal regions CA1 and CA2. We combined this with electrophysiological studies on the role of Kv2 channel function on AP characteristics in CA1 versus CA2 PNs by blocking Kv2 channels with GxTX, a selective blocker of Kv2 channels.

## Materials and Methods

### Antibodies

See details of antibodies used on Table 1.

**Table 1.** Antibodies used in this study

Antibody name	Species/isotype/immunogen	Manufacturer information	Concentration used
AMIGO-1, anti-AMIGO-1 rabbit pAb	Raised against aa 394–492 of mouse AMIGO-1 (cytoplasmic C-terminus).	Trimmer Lab. Rabbit 28330 RRID:AB_2571515	1:400 dilution of affinity purified pAb, concentration unknown
L98/12, anti-AMIGO-1 mouse IgG1 mAb	Raised against aa 28–370 of mouse AMIGO-1 (extracellular N-terminus).	Trimmer lab. RRID:AB_2571516	1:3 dilution of tissue culture supernatant, concentration unknown
K89/34, anti-Kv2.1 mouse IgG1 mAb	Raised against aa 837–853 of rat Kv2.1.	Trimmer lab. NeuroMab catalog 73-014 RRID:AB_10672253	5 µg/ml purified mAb
L61C/30, anti-Kv2.1 mouse IgG1 mAb	Raised against aa 595–616 of rat Kv2.1.	Trimmer lab. RRID:AB_2532100	1:5 dilution of tissue culture supernatant, concentration unknown
N372B/1, anti-Kv2.2 mouse IgG1 mAb	Raised against aa 717–907 of rat Kv2.2. Binds within aa 764–907. Species reactivity with mouse, rat, ferret, macaque and human	NeuroMab catalog 73–369, RRID:AB_2315869	1:3 dilution of tissue culture supernatant, concentration unknown
N372B/60, anti-Kv2.2 mouse IgG2b mAb	Raised against aa 717–907 of rat Kv2.2. Binds within aa 764–907. Species reactivity with mouse and rat	NeuroMab catalog 73–360, RRID:AB_2315867	1:10 dilution of tissue culture supernatant, concentration unknown
N372C/51, anti-Kv2.2 mouse IgG1 mAb	Raised against aa 717–907 of rat Kv2.2. Binds within aa 717–763 Species reactivity with mouse and rat	NeuroMab catalog 73–358, RRID:AB_2315865	1:2 dilution of tissue culture supernatant, concentration unknown
N133/21, anti-RGS14 mouse IgG2a mAb	Raised against aa 1–544 of rat RGS14.	NeuroMab catalog 73–170, RRID:AB_10698026	1:10 dilution of tissue culture supernatant, concentration unknown

Details of the polyclonal (pAb) and monoclonal (mAb) Abs used in this study.

### C57/BL6J mice

All procedures involving C57/BL6J mice were approved by the University of California Davis Institutional Animal Care and Use Committee and were performed in strict accordance with the Guide for the Care and Use of Laboratory Animals of the NIH. All C57/BL6J mice were maintained under standard light-dark cycles and allowed to feed and drink ad libitum. C57BL/6J were purchased from The Jackson Laboratory (RRID:IMSR_JAX:000664). Both male and female 12-week-old C57BL/6J were used.

### Preparation of C57/BL6J mouse brain sections for immunohistochemistry

Eight adult C57/BL6J mice (four females and four males) were deeply anesthetized with 90 mg/kg Na-pentobarbital salt (Sigma) in 0.9% NaCl solution through intraperitoneal injections, followed by boosts as needed. Once mice were completely anesthetized, they were transcardially perfused with a brief prefix wash with 4.5 ml of ice cold PBS [150 mM NaCl, 10 mM sodium phosphate buffer (PB), pH 7.4] containing 10 U/ml heparin, followed by an ice-cold fixative solution of 4% formaldehyde (freshly prepared from paraformaldehyde) in 0.1 M sodium PB, pH 7.4, using a volume of 1 ml fixative solution per gram of mouse weight. Following perfusions, brains were removed from the skull and cryoprotected in 10% sucrose, 0.1 M PB overnight at 4°C, then transferred to a solution of 30% sucrose, 0.1 M PB until they sank to the bottom of the tube (24–48 h). Following cryoprotection, all brains were frozen, and cut on a freezing stage sliding microtome (Richard Allen Scientific) to obtain 30-µm-thick sections. Sections were collected in 0.1 M PB and processed for immunohistochemistry as free-floating sections.

### *Amigo-2/Cre* mice

All procedures involving *Amigo-2/Cre* mice were approved by the Institutional Animal Care and Use Committee at Columbia University and the New York State Psychiatric Institute. *Amigo-2/Cre* mice were maintained as hemizygotes on the C57BL/6J background. Three male and three female 8-week-old mice were used for stereotaxic injections. GFP expression in CA2 neurons was achieved by stereotaxic injection of a Cre-dependent adeno-associated virus (AAV) expressing eGFP into *Amigo-2/Cre* mice, as previously described (Hitti and Siegelbaum, 2014). The AAV2/5-hSyn-DIO-eGFP-WPRE-hGH virus was obtained from the University of Pennsylvania Vector Core. In brief, for stereotaxic surgery mice were anesthetized with continuous isoflurane delivery. Temperature and anesthesia depth were monitored periodically. To target CA2, mice were injected bilaterally with 180 nl of virus at coordinates −1.6 AP, ±1.6 ML, −1.7 DV or −1.7 AP, ±1.9 ML, −1.8 DV relative to bregma using a Nanoject II (Drummond Scientific) auto-nanoliter injector. Mice recovered for three weeks to allow for adequate viral expression. Mice were then deeply anesthetized with isoflurane (5%) and perfused transcardially with 4.5 ml ice-cold 0.1 M PBS buffer followed by 4% formaldehyde (freshly prepared from paraformaldehyde) in PBS, using a volume of 1 ml fixative solution per gram of mouse weight. After decapitation, brains were removed and cryoprotected in 30% sucrose in 0.1 M PBS overnight at 4°C before freezing.

### Multiplexed fluorescence immunohistochemistry

Multiplex immunofluorescence labeling of mouse brain sections was performed essentially as described (Manning et al., 2012). In brief, free-floating sections were washed 3× in 0.1 M PB and 10 mM sodium azide at room temperature with slow agitation. All subsequent incubations and washes were at room temperature with slow agitation. Sections were incubated in blocking buffer (10% goat serum in 0.1 M PB, 0.3% Triton X-100, and 10 mM sodium azide) for 1 h. Immediately after blocking, sections were incubated with primary antibody combinations (diluted in blocking buffer) for 2 h. Following incubation, sections were washed 3 × 10 min each in 0.1 M PB, and incubated for 1 h with affinity-purified goat anti-rabbit and/or goat anti-mouse IgG-subclass-specific secondary antibodies conjugated to Alexa Fluors (ThermoFisher) and diluted in blocking buffer. Sections were labeled with the DNA-specific dye Hoechst 33258 during the secondary antibody step. After 3 × 10 min washes in 0.1 M PB, sections were mounted and dried onto gelatin-coated slides, treated with 0.05% Sudan Black (EM Sciences) in 70% ethanol for 1.5 min (Schnell et al., 1999), extensively washed in water, and mounted with Prolong Gold (ThermoFisher). All immunolabeling reported is representative of at least five animals (biological replicates), unless otherwise noted. Brain sections from all biological replicates within each experiment were labeled, treated, and mounted in parallel.

### Imaging and image analysis

All images were acquired on a Zeiss AxioObserver Z1 microscope with an X-Cite 120 lamp as the fluorescent light source and equipped with an AxioCam MRm digital camera. Low-magnification wide-field imaging was done using a 10×/0.5 NA Fluar objective, and images were reconstructed as tiled mosaics using Axiovision 4.8.2 acquisition software (Carl Zeiss MicroImaging, RRID: SciRes_000111). High-magnification optical sections were acquired using an ApoTome structured illumination system (Carl Zeiss MicroImaging) with a 63×/1.40 NA plan-Apochromat oil immersion objective. ApoTome z-stacks were acquired and processed with Axiovision 4.8.2 acquisition software (Carl Zeiss MicroImaging, RRID: SciRes_000111). All brain sections within a given experiment and immunolabeled with the same antibody cocktail were imaged under the same conditions (objective, exposure time, lamp settings, etc.). Image processing was performed in Axiovision (Carl Zeiss MicroImaging), ImageJ (NIH) and MATLAB (MathWorks). All panels in a given figure were imaged and treated identically, unless otherwise noted. Low-magnification wide-field mosaics and high-magnification ApoTome z-stacks were opened for analysis as raw image files in ImageJ (NIH) using the Bio-Formats library importing plugin (Linkert et al., 2010). All statistical analyses of immunolabeling were performed in Prism (GraphPad).

Linescans were used to measure labeling intensity across the different regions of CA (CA1 to CA2 to CA3) in low-magnification (10×) mosaic images of hippocampus. Linescans were 18 µm (28 pixels) thick, and data points reflected the mean intensity values (mean of 28 pixels) per area unit. Linescans were drawn across CA1 to CA2 to CA3 in stratum pyramidale (s.p.). Values from multiple immunolabels and of Hoechst dye were simultaneously measured from the same linescan. Background levels for individual labels were measured from no primary controls and mathematically subtracted from linescan values. Intensity values for each linescan and for individual antibody labels were normalized to their own average intensity in the CA region with the highest level of immunolabeling. Linescans from different brain sections (and from different animals) were aligned based on RGS14 labeling and the normalized values across different linescans were averaged. Mean fluorescence intensity values in high-magnification (63×) images were quantified by ROI selections. Individual neurons were identified by Hoechst staining. CA2 PNs were identified by RGS14 labeling. ROI selections were drawn in single optical sections, which show signal from only one neuronal layer in the Z plane. Each ROI was drawn around the Kv2-labeled area clearly seen in the periphery of the neurons using the “polygon selection tool” in ImageJ. Intensity values were normalized to the average value of CA1 neurons.

### Electrophysiology slice preparation

C57BL/6J mice were housed and euthanized in accordance with Université Paris Descartes ethics committee approval. C57BL/6J male mice (8–10 weeks old) were anesthetized with ketamine/xylazine and isoflurane. Mice were intracardially perfused with oxygenated cutting solution containing 93 mM NMDG, 2.5 mM KCl, 1.25 mM NaH_2_PO_4,_ 30 mM NaHCO_3_, 20 mM HEPES acid, 25 mM glucose, 2 mM thiourea, 5 mM Na-ascorbate, 3 mM Na-pyruvate, 0.5 mM CaCl_2_, 10 mM MgCl_2_, and 93 mM HCl. Hippocampi were removed and placed upright into an agar mold and 400-μm-thick transverse slices were cut with a vibratome (Leica VT1200S) in ice-cold solution and transferred to 30°C ACSF (125 mM NaCl, 2.5 mM KCl, 10 mM glucose, 26 mM NaHCO_3_, 1.25 mM NaH_2_PO_4_, 2 mM Na pyruvate, 2 mM CaCl_2_, and 1 mM MgCl_2_) for 30 min and kept at room temperature for at least 1.5 h before recording. Cutting and recording solutions were both saturated with 95% O_2_ and 5% CO_2_, pH 7.4.

### Electrophysiological recordings and analysis

Whole-cell recordings were obtained with an Axon 700B amplifier and Digidata 200 ADDA converter (Molecular Devices). A slice scope (Scientifica) equipped with an IR LED and Dodt contrast was used to visualize the slices, which were held into the recording chamber with a platinum and nylon harp. ACSF was perfused through the recording chamber at 3 ml/min. All experiments were performed at 33°C. Recordings were performed with a patch pipette (3 MΩ pipette resistance) containing 135 mM K-methylsulfate, 5 mM KCl, 0.1 mM EGTA-Na, 10 mM HEPES, 2 mM NaCl, 5 mM MgATP, 0.4 mM Na_2_GTP, 10 mM Na_2_ phosphocreatine, and 5 µM biocytin (pH 7.2; 280–290 mOsm). The liquid junction potential was ∼2 mV and membrane potentials were corrected *post hoc*. Series resistance (typically 12–15 MΩ) was carefully monitored in voltage clamp mode by the application of a 100-ms −5-mV step. The membrane resistance (R_M_), membrane capacitance (C_M_) and resting membrane potential (RMP) were measured for each cell within 5 min after break-in. In current clamp, the amplifier circuitry (Axon 700B, Molecular Devices) was used to compensate the bridge balance, which was carefully monitored during experiments. Cells with >10% change in series resistance or leak were excluded from analysis. For all experiments involving measurements of AP firing, current was injected to keep the membrane potential at −70 mV for both CA1 and CA2 PNs. GxTX (Smartox Biotechnology) was dissolved in 0.1% BSA and aliquoted as 100 µM stocks. Aliquots were promptly thawed before use and predissolved in ACSF before bath application. For every cell that we recorded, we allowed the cell’s intrinsic properties to stabilize and then waited 9 ± 2 min before applying GxTX. Before GxTX application, we performed the recording protocol every 2 min to measure AP firing properties to ensure that cell firing was not changing with time. Changes in CA1 PN firing properties were seen within 4 min of GxTX application. However, all recordings from GxTX-treated CA1 and CA2 PNs were performed 8 min after GxTX application to ensure the completeness of the effect of the toxin. We used pClamp10 software for data acquisition and AxographX and Origin Pro for data analysis and statistical testing. All analyses of AP shape and after-hyperpolarization potential (AHP) were performed as described by Liu and Bean (Liu and Bean, 2014). For both CA1 and CA2 PNs, the AHP was quantified by measuring the most hyperpolarized potential between APs. Statistical comparisons were performed using Student’s *t* test, two-way ANOVA with repeated measure when appropriate. Results are reported as mean ± SEM.

## Results

### Abrupt changes in Kv2 channel subunit expression at the CA1-CA2 boundary

Previous immunohistochemistry studies have described the expression patterns of the Kv2.1 and Kv2.2 α subunits in regions CA1 and CA3 of rodent hippocampus (Hwang et al., 1993; Maletic-Savatic et al., 1995; Sarmiere et al., 2008; Kirizs et al., 2014; Mandikian et al., 2014), and have noted high-level expression of Kv2.1 in CA1 PNs relative to CA3. However, these studies did not specifically address the expression of Kv2 channel subunits across the boundary between regions CA1 and CA2.

**Figure 1.**
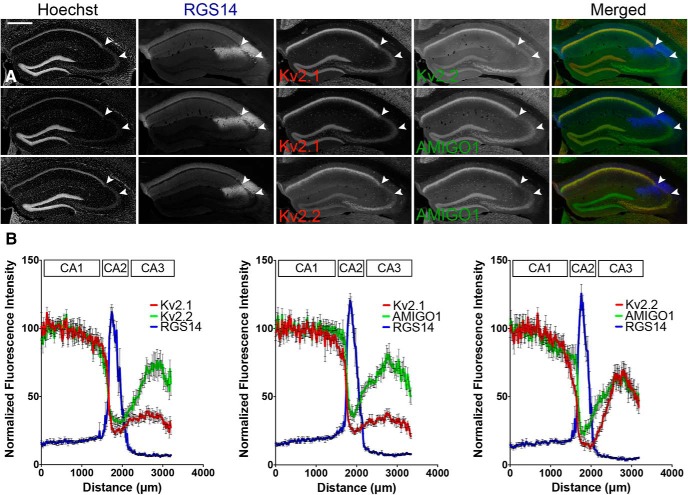
The distribution of Kv2 channel α and auxiliary subunit immunolabeling in s.p. changes at the CA1-CA2 boundary, and again within region CA3 of mouse hippocampus. ***A***, Representative low-magnification (10×, wide-field mosaic) images of C57BL/6J mouse coronal brain sections immunelabeled for combinations of Kv2.1, Kv2.2, AMIGO-1, and the CA2 marker RGS14. Arrowheads indicate the boundaries of region CA2 based on RGS14 immunolabeling. Scale bar, 500 µm. ***B***, Quantification of mean fluorescence intensity from linescans across s.p. of regions CA1, CA2, and CA3. Values are normalized to the maximum average intensity (*n* = 8 mice).

To determine the relative expression of Kv2.1, Kv2.2, and their auxiliary subunit AMIGO-1 in region CA1 versus CA2, we used multiplex fluorescence immunohistochemistry to label brain sections from C57BL/6J mice with KO-validated antibodies against the individual members of the Kv2 family (Mandikian et al., 2014; Bishop et al., 2015) together with markers for region CA2, including a monoclonal antibody against the CA2-specific marker RGS14 (Lee et al., 2010; Kohara et al., 2014). As shown in Figure 1, immunolabeling for each of the Kv2 channel α and AMIGO-1 auxiliary subunit antibodies exhibits a substantial reduction in labeling intensity at or near CA1-CA2 boundary, delineated by RGS14 labeling. We next extended these observations to determine how expression varies across the full extent of regions CA1, CA2, and CA3 of mouse hippocampus. Images representative of those used for quantitation are shown in Figure 1*A*, and the quantification of fluorescence intensity determined by linescans across s.p. of CA1-CA3 is shown in Figure 1*B*. As previously reported, RGS14 labeling is concentrated in the relatively small area between regions CA1 and CA3 (Lee et al., 2010; Kohara et al., 2014). This RGS14-positive CA region is characterized by large cell bodies and lower neuronal density that typifies CA2 (Lorente de Nó, 1934). Kv2.1 immunolabeling is highest in s.p., in accordance with its restricted subcellular localization on the soma, proximal dendrites and axon initial segment. The highest Kv2.1 immunolabeling intensity corresponds to region CA1, with a pronounced decrease at the CA1-CA2 boundary, as demarcated by the site of the sharp increase in RGS14 labeling (Fig. 1). The lower immunolabeling levels of Kv2.1 in CA2 were preserved across CA3 (Fig. 1). Immunolabeling for the Kv2.2 α subunit was also highest in CA1 and dropped sharply at the CA1-CA2 border. Similar to the pattern seen for Kv2.1 and Kv2.2, immunolabeling for the AMIGO-1 auxiliary subunit also decreased at the CA1-CA2 boundary (Fig. 1). Qualitatively similar results were obtained with independent antibodies with distinct binding sites on each Kv2 channel subunit (data not shown), supporting that these patterns of immunolabeling were reflective of differences in expression levels and not immunoreactivity, per se. In contrast to Kv2.1, Kv2.2, and AMIGO-1 exhibited a graded expression across region CA3 (Fig. 1), with lower labeling in distal CA3 (nearest CA2) and higher immunolabeling in proximal CA3 (toward the dentate gyrus).

**Figure 2.**
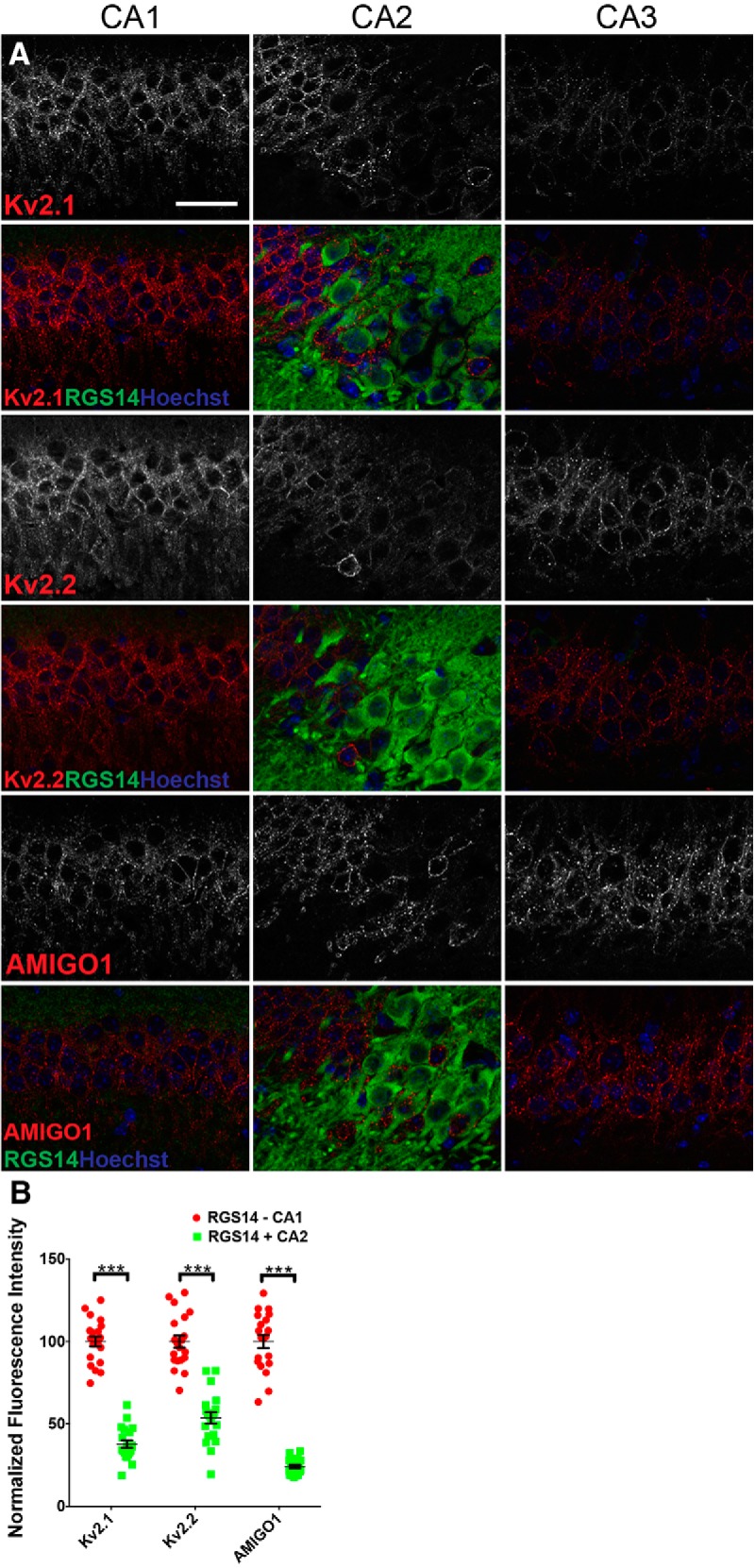
Individual CA2 PNs have reduced levels of Kv2 channel α and auxiliary subunit immunolabeling compared to CA1 neurons. ***A***, High-magnification (63×) representative images of C57BL/6J mouse coronal brain sections immunolabeled for Kv2.1, Kv2.2, AMIGO-1, and RGS14. Single optical z-section images (ApoTome Zeiss). Scale bar, 35 µm. ***B***, Quantification of mean fluorescence intensity from ROIs corresponding to individual RGS14 + and RGS14 – PNs. Values are normalized to average values in CA1 PNs. (*n* = 4 mice). Error bars show SEM. Asterisks denote samples exhibiting significant differences (*p* < 0.001, unpaired *t* test).

The density of PNs decreases substantially moving from CA1 to CA2 (Lorente de Nó, 1934). In our experiments, we found that the density of Hoechst nuclear labeling is indeed lower in CA2 compared to CA1. As such, it is possible that the differences in the overall intensity of immunolabeling for Kv2 channel subunits seen at the CA1-CA2 border is simply a reflection of the lower density of PNs in CA2, as opposed to lower expression of these Kv2 α and auxiliary subunits in individual PNs of region CA2. To address this, we next performed high-magnification imaging with optical sectioning to define the immunolabeling on a cell-by-cell basis, and determine differences in expression between RGS14 positive and negative neurons within the CA regions. As shown in the representative images depicted in Figure 2*A*, the RGS14 negative PNs in CA1 proper and at the CA1-CA2 boundary have the highest labeling intensity for Kv2.1, Kv2.2, and AMIGO-1, whereas RGS14 positive neurons within the CA1-CA2 boundary zone, and within CA2 proper, have relatively low levels of such immunolabeling. The differences in immunolabeling intensities observed between CA1 and CA2 PNs are statistically significant as seen by the quantification of fluorescence intensity performed on individual PNs (Fig. 2*B*). Similar to what is seen in the low-magnification images, region CA3 contains neurons that have a lower intensity of Kv2.1 immunolabeling than seen in CA1, but with levels of Kv2.2 and AMIGO-1 immunolabeling in proximal CA3 approaching those seen in CA1 (Fig. 2*A*).

**Figure 3.**
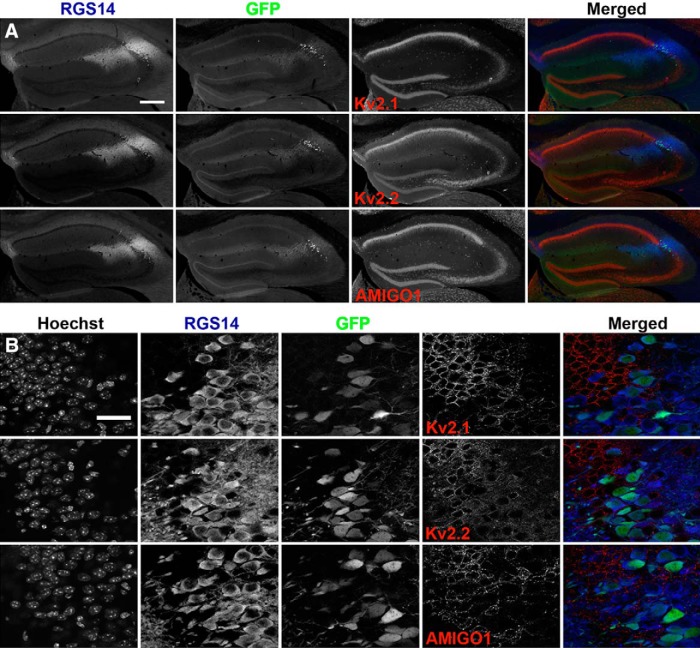
*Amigo-2/Cre* GFP-positive CA2 PNs have reduced levels of Kv2 channel α and auxiliary subunit immunolabeling. ***A***, Representative low-magnification (10×, wide-field mosaic) images of *Amigo-2/Cre* mice expressing GFP in CA2 PNs. Coronal brain sections from five mice were immunolabeled for Kv2.1, Kv2.2, AMIGO-1, and RGS14. Scale bar, 500 μm. ***B***, High-magnification (63×) representative images of *Amigo-2/Cre* mice expressing GFP in CA2 PNs. Single optical z-sections images (ApoTome Zeiss) were obtained from five *Amigo-2/*Cre mice immunolabeled for Kv2.1, Kv2.2, AMIGO-1, and RGS14. Scale bar, 35 μm.

To confirm results obtained using RGS14 immunolabeling to define region CA2, we next used the distinct CA2 marker AMIGO-2. *Amigo-2/Cre* mice expressing GFP in CA2 PNs (Hitti and Siegelbaum, 2014) were used to distinguish CA2 PNs. Brain sections from these mice were immunolabeled for Kv2.1, Kv2.2, or AMIGO-1, as well as for RGS14. Figure 3*A* shows representative low-magnification images of the hippocampus that show a high degree of concordance between GFP-positive and RGS14-positive neurons, as was previously demonstrated for this transgenic mouse line (Hitti and Siegelbaum, 2014). The overall pattern of Kv2.1, Kv2.2, and AMIGO-1 immunolabeling in brain sections from these mice (Fig. 3) is indistinguishable from that seen in C57BL/6J mice (Fig. 1). Moreover, the CA2 boundary demarcated by GFP expression corresponds well to the region in which the differences in Kv2.1, Kv2.2, and AMIGO-1 immunolabeling differ across the CA regions. Higher magnification imaging with optical sectioning in the CA1-CA2 boundary region revealed lower levels of Kv2 channel subunit immunolabeling in individual GFP-positive PNs than in GFP-negative neurons (Fig. 3*B*). These observations were comparable to those seen when RGS14 immunolabeling was used to define CA2 neurons in C57BL/6J mice. Taken together, these results show that the expression of Kv2 α and auxiliary subunits drops sharply at the CA1-CA2 border, and remains low throughout CA2 compared to CA1. Moreover, they show that Kv2.2 and AMIGO-1, but not Kv2.1, exhibit a gradual increase in CA3, with higher levels of expression in proximal versus distal CA3.

### Kv2 channels play a prominent role in determining AP properties in CA1 but not CA2 PNs

**Figure 4.**
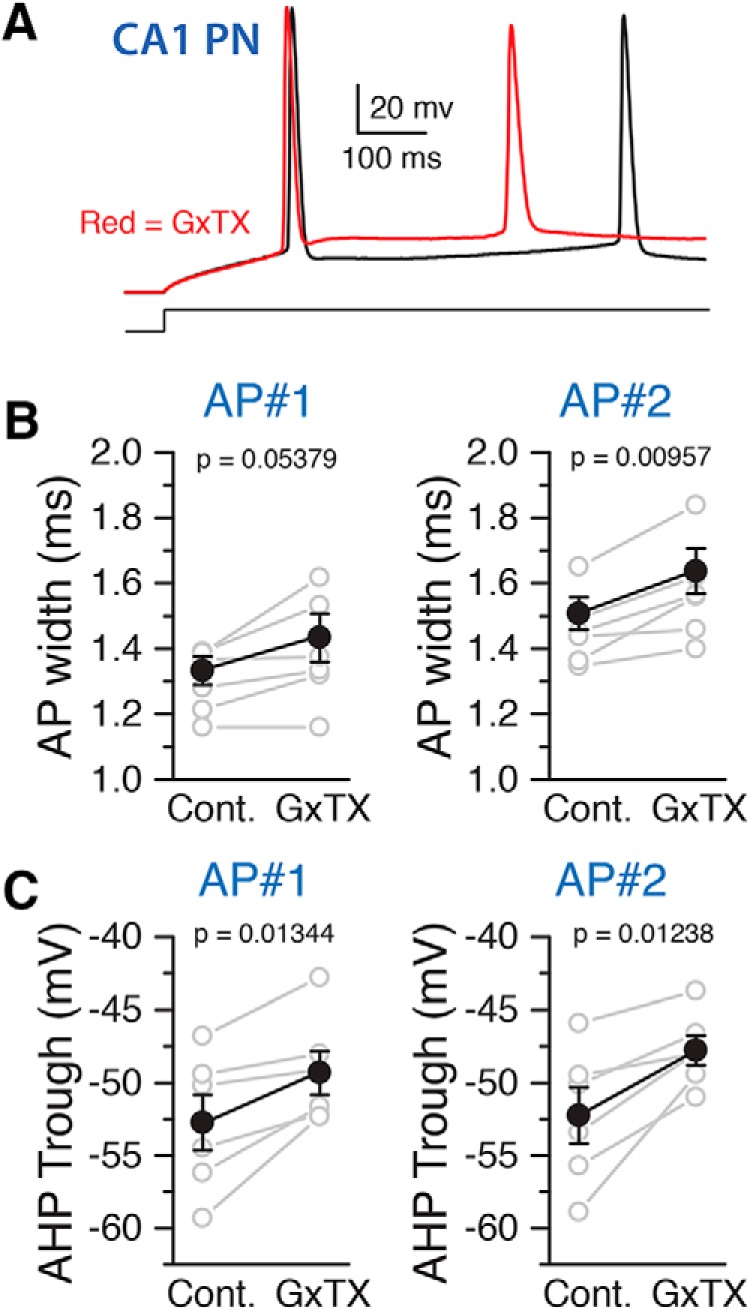
GxTX effects on AP characteristics in CA1 PNs. Bath application of 100 µM GxTX altered the AP shape and AHP of CA1 PNs. ***A***, Example traces of an AP in a CA1 PN as recorded in whole-cell current clamp configuration in response to a current injection of 260 pA before (black) and after (red) the application of 100 nM GxTX. ***B***, Summary graph showing the measured AP widths for the 1st and 2nd AP before and after the application of GxTX. Gray symbols are individual cells, black is the mean. ***C***, Summary graph showing the minimum AHP trough potential following the 1st and 2nd AP before and after GxTX application. Gray, individual points, black, mean. Error bars show SEM.

Our immunolabeling results indicate that the expression levels of Kv2 channel subunits are substantially lower in CA2 PNs relative to those in CA1. A previous study in acutely dissociated mouse CA1 neurons demonstrated that selectively blocking Kv2 channels with 100 nM GxTX blocks 60–80% of delayed rectifier currents, and alters initial firing frequency and AHP trough (Liu and Bean, 2014). Here, based on the expression analyses above, we performed a comparison of Kv2 channel function in CA1 versus CA2 neurons. We performed electrophysiology on acute mouse hippocampal slices and recorded from CA1 and CA2 PNs in whole-cell current clamp mode, before and after bath application of 100 nM GxTX. We first validated the use of GxTX application in acute hippocampal slices. Changes in CA1 PN firing properties were seen within 4 min of GxTX application. However, all recordings from GxTX-treated CA1 and CA2 PNs were performed 8 min after GxTX application to ensure the completeness of the effect of the toxin. We demonstrated that in this preparation, GxTX application has a similar effect on CA1 PN AP firing properties that has previously been described in acutely dissociated mouse neurons (Liu and Bean, 2014). While we did not detect a significant increase in the first AP width following block of Kv2 channels, we observed a significant increase in the width of the second AP (Fig. 4). Furthermore, the block of Kv2 channels resulted in a significant increase in the AHP trough potential following both the first and second APs (Fig. 4).

**Figure 5.**
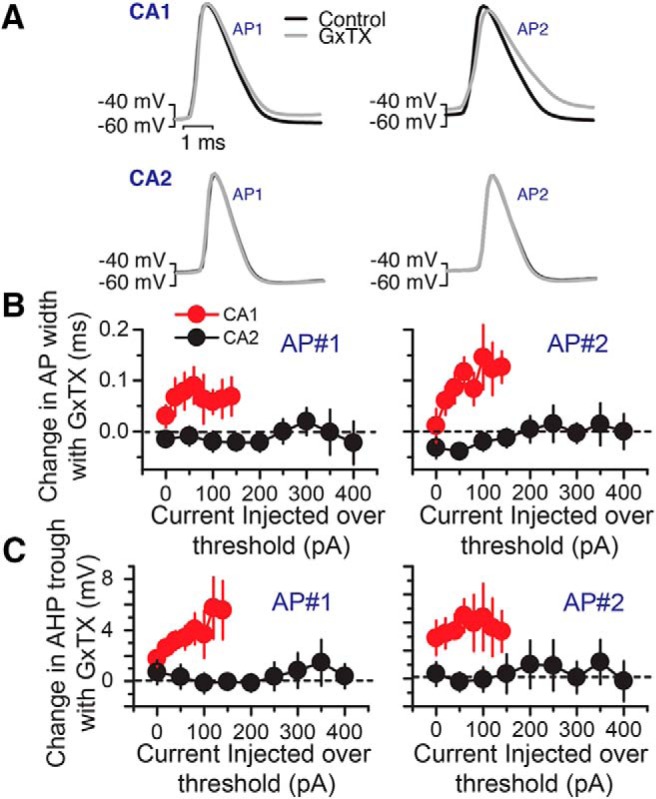
GxTX has different effects on CA1 and CA2 PN AP properties. ***A***, Example traces from CA1 and CA2 PNs recorded in whole-cell current clamp mode in response to current injections of different duration and amplitude before (black) and after (gray) the application of 100 nM GxTX. ***B***, Summary graph showing the change in AP width following GxTX application as a function of current injection over threshold for CA1 (red) and CA2 (black) PNs. Data are presented for both the 1st and 2nd AP. ***C***, Summary graph of the change in minimal potential of the AHP trough following GxTX application as a function of current injection for the 1st and 2nd AP.

We postulated that because our immunolabeling experiments reveal a sharp drop in immunolabeling intensity for Kv2 α and auxiliary subunits at the CA1-CA2 border, CA2 PNs likely do not express substantial levels of Kv2 channels relative to CA1 PNs. Thus, the application of GxTX application would have little or no effect on CA2 PN AP firing properties. We tested this hypothesis by performing whole-cell current clamp recordings in both CA1 and CA2 PNs. As shown in Table 2, the CA1 and CA2 PNs in our slice preparations exhibited a difference, while not significant, in RMPs (*p* = 0. 2), and significant differences in R_M_ (*p* = 0.04), and C_M_ (*p* = 0.03), consistent with what has been previously described (Chevaleyre and Siegelbaum, 2010). Thus, to determine what effect GxTX application had on AP shape and membrane excitability, we first determined the AP threshold and injected 1-s-long steps of depolarizing current (Fig. 5). While we consistently saw an increase in AP width in CA1 PNs for the 2nd AP, we never observed such a change in AP shape in CA2 PNs. Furthermore, when examining changes in AHP trough following GxTX application, we consistently observed a substantial change in the minimal potential of the AHP trough on GxTX treatment in CA1 PNs, but no change in CA2 PNs (Fig. 5*C*), even following injections of current 300 pA over AP threshold.

**Table 2.** Intrinsic membrane properties of CA1 and CA2 PNs

PN type	RMP (mV)	R_M_ (MΩ)	C_M_ (pF)
CA1 (*N* = 6)	−70.2 ± 1.3	84.8 ± 13.8	151.4 ± 38.6
CA2 (*N* =5)	−73.1 ± 1.6	49.1 ± 3.7	316.3 ± 56.3

**Figure 6.**
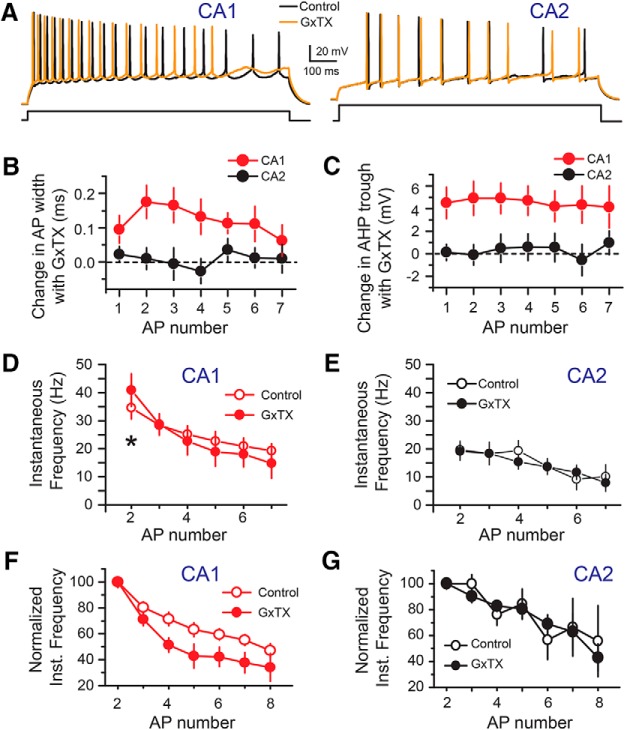
GxTX has different effects on repetitive firing in CA1 versus CA2 PNs. ***A***, Example traces from CA1 and CA2 PNs recorded in whole-cell current clamp mode in response to a 1-s long current injection of 460 pA before (black) and after (orange) the application of 100 nM GxTX. Note how GxTX altered several properties of CA1 PN AP firing but had little to no effect on CA2 PNs. ***B***, Summary graph showing the change in AP width with GxTX application as a function of AP number. Note the consistent change in AP width in CA1 whereas no increase was observed with CA2. ***C***, Summary graph of the change in minimal potential of the AHP trough following GxTX application as a function of AP number. ***D***, The instantaneous frequency of AP firing in CA1 PNs before (open circles) and after (closed circles) the application of GxTX. The block of Kv2 channels significantly increased the instantaneous frequency of the first two APs, but had no significant effect on the subsequent instantaneous firing frequencies. ***E***, The instantaneous frequency of AP firing in CA2 PNs before (open circles) and after (closed circles) the application of GxTX. ***F***, The instantaneous frequency normalized to the first AP, to illustrate the changes in adaptive AP firing for CA1 PNs before (open circles) and after (closed circles) the application of GxTX. Current injection step was around 300 pA over AP threshold. ***G***, Summary graph of the normalized instantaneous frequency for CA2 PNs before (open circles) and after application of GxTX (filled circles) as a function of AP number. Current injection step was 500 pA over AP threshold. Error bars show SEM.

We also examined the change in AP width and AHP trough as a function of AP number before and after application of GxTX for CA1 and CA2 PNs. For CA1 PNs, there is a consistent increase in AP width for the second and subsequent APs (Fig. 6*A*,*B*), whereas for CA2 PNs, no significant change was observed following GxTX application. Likewise, we observed a consistent increase in AHP trough for all APs measured in area CA1 following GxTX application, but no effect in area CA2 (Fig. 6*C*). Lastly, we examined how blockade of Kv2 current by GxTX application altered the instantaneous firing frequency of CA1 and CA2 PNs. We found that when comparing the instantaneous frequency of the first two APs, application of GxTX significantly increased the AP firing frequency between the first and second APs in CA1 PNs (control: 34.6 ± 4.0 Hz, GxTX: 40.8 ± 3.8, *n* = 6, *p* = 0.03). This is consistent with the change in AHP trough that we observe, indicating that Kv2 channels are active in controlling the initial burst firing and membrane excitability in CA1 PNs (Fig. 6*D*). When considering the dynamic firing properties of CA1 PNs, we found that blocking Kv2 channels in CA1 PNs increases adaptive AP firing (Fig. 6*F*), and could prevent AP firing following ≈800 ms of current injection (Fig. 6*A*). In contrast, we observed no detectible effect on CA2 PN instantaneous frequency following application of GxTX (Fig. 6*E*,*G*). Together, these results show that Kv2 channels play a prominent role in determining the firing properties of CA1 but not CA2 PNs.

## Discussion

Delayed rectifier Kv channels of the Kv2 family are expressed in PNs in s.p. of the hippocampus (Hwang et al., 1993; Maletic-Savatic et al., 1995; Kuja-Panula et al., 2003; Mandikian et al., 2014). The studies presented here showed substantial differences in expression of Kv2 channel α and auxiliary subunits precisely at the CA1-CA2 boundary, with much lower levels in s.p. of CA2 compared to those in CA1. We show that these lower levels of immunolabeling are not simply due to the lower cell density in CA2 versus CA1, but are reflective of differences in immunolabeling for these Kv2 channel subunits within individual CA2 PNs, as quantified using high-magnification optical section imaging. The difference in the levels of Kv2 channel immunolabeling in CA1 versus CA2 PNs was observed in brain tissue from both C57BL/6J and *Amigo-2/Cre* mice. That similar results were obtained with at least two monoclonal antibodies with independent non-overlapping binding sites on each subunit, and also with polyclonal antibody preparations, strongly supports that this immunolabeling reflects the levels of expression of the individual subunits in these neurons. The cellular pattern of immunolabeling obtained here is generally consistent with previously published immunolabeling results, and with *in situ* hybridization studies posted by the Allen Brain Atlas. However, our results provide higher resolution information that this change in Kv2 channel subunit expression occurs precisely at the CA1:CA2 boundary, which these other sources could not provide due to the lack of specific molecular definition of region CA2.

Consistent with these immunolabeling results is the overall lack of an effect of GxTX on AP characteristics in CA2 PNs, relative to the robust and diverse effects of this neurotoxin on APs in CA1 neurons. The results presented here, obtained from CA1 PNs in intact hippocampal slice preparations, are in good agreement with previous results from acutely dissociated CA1 neurons (Liu and Bean, 2014). Our results demonstrate a prominent role for Kv2 channels in CA1 PNs, where they are highly expressed on the soma, proximal dendrites, and axon initial segment, in determining membrane excitability, and the shape and frequency of APs. In contrast, we show that unlike in CA1 PNs, APs in CA2 PNs lack any demonstrable response to GxTX treatment, consistent with the significantly reduced expression of Kv2 channels observed in these PNs relative to those in CA1. CA2 PNs are distinct in lacking the afterhyperpolarizing trough seen in CA1 neurons (Chevaleyre and Siegelbaum, 2010), a characteristic that has been attributed to Kv2 channels (Liu and Bean, 2014). The substantially reduced expression of Kv2 channels likely confers a distinct input-output relationship to CA2 neurons, that, when combined with their unique network connectivity, would underlie their fundamentally different function in hippocampal information processing.

Our experiments show that Kv2 currents clearly contribute to the AP width and AHP of CA1 PNs. Thus, in CA1 PNs, by controlling the AP width, these channels are likely acting to control neuronal excitability during periods of high activity by limiting calcium influx during AP firing, as has been demonstrated in hippocampal slice cultures treated with antisense oligonucleotides targeting Kv2.1 (Du et al., 2000). Presynaptic neurotransmitter release paired with AP firing is a central component of long-term potentiation (Magee and Johnston, 1997). By their contribution to the AHP, we postulate that Kv2 currents may be contributing to CA1 PN repetitive firing and induction of long-term plasticity, as indicated by intact acute slice recordings of the scaeffer-collateral-CA1 synapse in Kv2.1 knockout mice (Speca et al., 2014). Thus, these channels both prevent hyperexcitability while simultaneously permitting plasticity in region CA1, a hippocampal area well established to central for memory formation and learning.

In contrast, it has been demonstrated by multiple groups that CA2 PNs are highly resistant to postsynaptic long-term potentiation (Zhao et al., 2007; Chevaleyre and Siegelbaum, 2010). While the consequence of this lack of plasticity in CA2 PNs is not fully understood, differences in intracellular signaling cascades (Lee et al., 2010) as well as calcium buffering (Simons et al., 2009) have been proposed to underlie this unusual property. Furthermore, differences in expression of numerous ion channels, including the absence of Kv2 channels, gives CA2 PNs very different properties compared to neighboring CA1. For instance, CA2 PNs have more hyperpolarized membrane potential and much lower R_M_ that CA1 PNs, likely due to the expression of voltage-gated and leak channels, making these cells less excitable than CA1 PNs in general. The integrative properties of CA2 PN dendrites have shown to be unique, allowing the propagation of distal dendritic input in a manner that is strikingly different from CA1 (Sun et al., 2014; Srinivas et al., 2017). It is clear that *in vivo*, CA2 PNs do not behave like CA1 PNs, with rapidly re-mapping place cells (Mankin et al., 2015) and the ability to encode location during immobility (Kay et al., 2016). Thus, there remains much to be learned about CA2 PN physiology and function.

We also note that there is a gradient of Kv2.2 and AMIGO-1, but not Kv2.1, from low in the distal region of CA3 (adjacent to CA2) and progressively higher toward the proximal region of CA3 (closer to the dentate gyrus). The elevated expression of Kv2.2, but not Kv2.1, in proximal CA3 may confer on these neurons properties distinct from distal CA3 and CA2, which lack prominent Kv2 channel expression, and CA1, which expresses high levels of both Kv2.1 and Kv2.2. This may impact the plasticity of Kv2 channel function in these neurons, given the robust phosphorylation-dependent regulation of Kv2.1 but not Kv2.2 (Bishop et al., 2015). Gradients in gene expression, connectivity and functionality across CA3 have been reported (Li et al., 1994; Thompson et al., 2008; Nakamura et al., 2013). Several Kv channels, such as Kv3.1, Kv3.2, Kv4.3, Kv5.1, and Kv10.1 have been shown to have a gradient of expression across CA3 at the mRNA level (Vega-Saenz de Miera, 2004; Thompson et al., 2008). The differences in ion channel expression across region CA3 presumably support the observed differences in intrinsic excitability and firing patterns in distal versus proximal CA3 PNs (Bragdon et al., 1986). Future studies employing GxTX may be useful to defining the specific role of the gradient of Kv2.2-containing delayed rectifier channels in these differences across region CA3, as shown here for the distinctions between PNs in regions CA1 and CA2.

## Synthesis

Reviewing Editor: Douglas Bayliss, University of Virginia School of Medicine Decisions are customarily a result of the Reviewing Editor and the peer reviewers coming together and discussing their recommendations until a consensus is reached. When revisions are invited, a fact-based synthesis statement explaining their decision and outlining what is needed to prepare a revision will be listed below.

The revised manuscript transferred to eNeuro has addressed, for the most part, all the minor comments from the previous review at Journal of Neuroscience. Although the recommendations from those reviews to use additional KV2 blockers and provide a voltage clamp assessment of blocker effects on KV2 currents were not heeded, the provided correlative data is strong and the hesitancy to expend more effort on examining a current that is evidently absent in CA2 neurons is understandable.

In light of this, the reviewing editor can recommend that the manuscript be accepted without further review, provided that a revision addresses some additional minor clarifications/corrections (listed below).

1. There seems to be some confusion with respect to the repetitive firing data presented in Fig. 6, and described on p. 15.

First, the exemplar record of Fig. 6A shows that GxTX increased firing frequency in the CA1 neuron (i.e., the APs in the yellow trace are, consistently and increasingly, left-shifted relative to the corresponding AP in the black trace, at least until they fade out). Yet, the data are described in the text (p. 15) as demonstrating that "application of GxTX substantially decreased the AP firing frequency in CA1 PNs."

This characterization appears to derive from the analysis presented in Fig. 6D, but this analysis also seems to be confused. How does one calculate an instantaneous frequency for each AP? Instantaneous frequency is typically derived from the reciprocal of the interspike interval, and thus requires two APs (as described in Liu & Bean, 2014, who used the first two spikes to calculate an initial firing rate during the step.)

So, in Fig. 6D, why does the normalized instantaneous frequency decrease more in GxTX through the pulse when in Fig. 6A the interspike intervals remain more closely spaced in GxTX (until the APs finally fade out)?

Moreover, the expectation is that a reduced AHP (more depolarized trough) would allow faster firing -- it would favor a shorter interspike interval, at least until progressive inactivation of sodium channels yields a fade-out. This is what the data in your exemplar trace indicates (Fig. 6A) ... but it is opposite of both the instantaneous frequency analysis (Fig. 6D) and the description in the text (p. 15).

This should be clarified.

2. The significance statement is overstated. It is not the case that "the differences in action potential firing properties in CA1 versus CA2 PNs can be attributed to Kv2 channels," at least not solely to Kv2 channels. Likewise, it is not possible to conclude that "Kv2 channel expression is the primary determinant of the distinct firing properties in CA1 versus CA2 PNs." These claims should be tempered.

3. I concur with the previous Reviewers point #9 regarding the awkward phrasing with the use of decrease, reduced, changes, etc. - there remain a few places in the manuscript where this should be corrected.

p. 12: "these patterns of immunolabeling were reflective of changes in expression levels ..." might better be "reflective of differences in expression levels"

"as opposed to a reduction in the expression of these Kv2 ..." to "as opposed to lower expression of these Kv2 ..."

"and determine changes in expression between RGS14 positive and negative neurons" to "and determine differences in expression between RGS14 positive and negative neurons"

p. 13: "Moreover, the CA2 boundary demarcated by GFP expression corresponds well to the region in which the changes in Kv2.1, Kv2.2 and AMIGO-1 immunolabeling differ across the CA regions." could instead say "Moreover, the CA2 boundary demarcated by GFP expression corresponds well to the region in which the differences in Kv2.1, Kv2.2 and AMIGO-1 immunolabeling are observed across the CA regions."

p. 14: "Our immunolabeling results indicate that the expression levels of Kv2 channel subunits are substantially reduced in CA2 PNs relative to those in CA1" to "Our immunolabeling results indicate that the expression levels of Kv2 channel subunits are substantially lower in CA2 PNs relative to those in CA1"

4. Please provide citation on p. 5 for "In the present study we used antibodies against the CA2 PN marker RGS14, an accepted molecular marker for CA2 PNs {refs} ..."
